# Density-Based Penalty Parameter Optimization on C-SVM

**DOI:** 10.1155/2014/851814

**Published:** 2014-07-07

**Authors:** Yun Liu, Jie Lian, Michael R. Bartolacci, Qing-An Zeng

**Affiliations:** ^1^Key Laboratory of Communication and Information Systems, Beijing Municipal Commission of Education, Beijing Jiaotong University, Beijing 100044, China; ^2^Information Sciences and Technology, Penn State University-Berks, Reading, PA 19610, USA; ^3^Department of Electronics, Computer and Information Technology, North Carolina Agricultural and Technical State University, Greensboro, NC 27411, USA

## Abstract

The support vector machine (SVM) is one of the most widely used approaches for data classification and regression. SVM achieves the largest distance between the positive and negative support vectors, which neglects the remote instances away from the SVM interface. In order to avoid a position change of the SVM interface as the result of an error system outlier, C-SVM was implemented to decrease the influences of the system's outliers. Traditional C-SVM holds a uniform parameter *C* for both positive and negative instances; however, according to the different number proportions and the data distribution, positive and negative instances should be set with different weights for the penalty parameter of the error terms. Therefore, in this paper, we propose density-based penalty parameter optimization of C-SVM. The experiential results indicated that our proposed algorithm has outstanding performance with respect to both precision and recall.

## 1. Introduction

Data classification algorithms, such as logistic regression (LR) [1–6] and support vector machine (SVM) [7–10], are crucial in many applications. SVM is a local optimum classification which pursues a maximum interval interface using a loss of the distance from the remote instances to the SVM interface [11–13]. The discriminant equation of the SVM model can be written as

(1) $$\begin{array}{c} Y=g\left(X;\omega \right)=\{\begin{array}{cc} 1 & \omega^{T}X+b>0\\ -1 & \omega^{T}X+b<0, \end{array} \end{array}$$

where *X* denotes an eigenvector of an arbitrary instance input and *x*_*i*_ is a concrete feature in an eigenvector in which *X* = {*x*_1_, *x*_2_,…, *x*_*m*_}. The model is trained with all positive instances of labels for which *Y* = 1 and the negative instances are trained with label *Y* = −1 in order to pursue the appropriate values for the parameters *ω* and *b*. Thus, for an unknown instance *X*_*i*_, it will be classified to a positive case when *ω* ^*T*^ *X*_*i*_ + *b* > 0, and vice versa.

Traditional SVM guarantees a strict classification that the classification model are constructed by the positive vectors with *ω* ^*T*^ *X*_*i*_ + *b* = 1, the negative vectors with *ω* ^*T*^ *X*_*i*_ + *b* = −1 and the SVM interface that *ω* ^*T*^ *X*_*i*_ + *b* = 0. Since all the positive instances hold the distances ≥1 and negative instances ≤1, this leads to the following problems: (1) in many datasets, positive and negative instances are interlaced which can not be classified under a regular kernel function; (2) a meticulous training may cause an overfitting phenomenon which to the maximum extent satisfies the classification in the training set by sacrificing the systematic performance for the data in the probe set; and (3) overtraining usually costs more computation. In order to solve these shortcomings, C-SVM is introduced to improve the adaptability of the traditional SVM model [14, 15]. In C-SVM model, coefficient *C* is used to control the tolerance of the systematic outliers which allows less outliers to exist in the opponent classification. Coefficient *C* is an empirical parameter which is usually worked out via a gird search process. C-SVM holds a uniform *C* for both positive instances and negative ones, which only satisfies the datasets with the similar distributions of each class. In LIBSVM, C-SVM model is improved by the number proportion of the positive instances to the negative ones [13, 14]; however the spatial distribution of the initial instances has not been involved in the model training process. In this paper, we aim to provide a better solution of the value of parameter *C*, thus under the same conditions it can achieve a relatively accurate classification result.

## 2. Traditional Model of SVM

According to (1), since *Y* is positive (or negative) when *ω* ^*T*^ *X*_*i*_ + *b* > 0 (or *ω* ^*T*^ *X*_*i*_ + *b* < 0), |*ω* ^*T*^ *X*_*i*_ + *b*| can be denoted by *s* = *Y*_*i*_(*ω* ^*T*^ *X*_*i*_ + *b*), where *s* is the distance between an arbitrary instance (*X*_*i*_, *Y*_*i*_) and the SVM interface. When seeking the appropriate *ω* and *b* in order to maximize the distance between the support vectors and the SVM interface, on the proportionally scale, the distance *s* will not change the values of *ω* and *b*. Thus, *s* can be presented by

(2) $$\begin{array}{c} s=Y_{i}\left({\left(\frac{\omega }{||\omega ||}\right)}^{T}X_{i}+\frac{b}{||\omega ||}\right). \end{array}$$

Normalizing the geometric interval to ||*ω*|| = 1, (2) can be subjected to

(3) max⁡ω,b⁡ ss.t. Yi(ωTXi+b)≥s i=1,2,3,…,n||ω||=1.

Since ||*ω*|| = 1 is not convex, for the max_*ω*,*b*_ *s*/||*ω*||, (3) is subject to

(4) min⁡ω,b⁡ 12||ω||2s.t. Yi(ωTXi+b)≥1 i=1,2,3,…,n.

Computing the minimum ||*ω*||^2^ under the condition of *Y*_*i*_(*ω* ^*T*^ *X*_*i*_ + *b*) ≥ 1, the Lagrangian function can be imported by

(5) $$\begin{array}{c} L\left(\alpha ,\omega ,b\right)=\frac{1}{2}{||\omega ||}^{2}+\overset{n}{\underset{i=1}{\sum }}\alpha_{i}\left(1-Y_{i}\left(\omega^{T}X_{i}+b\right)\right). \end{array}$$

The minimum *L*(*α*, *ω*, *b*) can be acquired by the derivation of the parameters *ω* and *b* such that

(6) ∂L(α,ω,b)∂ω=ω−∑i=1nαiYiXi=0⟹ω=∑i=1nαiYiXi∂L(α,ω,b)∂b=∑i=1nαiYi=0.

Integrating (5) and (6), we finally obtain

(7) L(α,ω,b)=12ωT∑i=1nαiYiXi+∑i=1nαi −∑i=1nαiYiωTXi−∑i=1nαiYib=−12ωT∑i=1nαiYiXi+∑i=1nαi−b∑i=1nαiYi=∑i=1nαi−12(∑i=1nαiYiXi)T∑i=1nαiYiXi−b∑i=1nαiYi=∑i=1nαi−12∑i=1,j=1nαiYiXiTαjYjXj−b∑i=1nαiYi=∑i=1nαi−12∑i=1,j=1nYiYjαiαjXiTXj−b∑i=1nαiYi.

Combining (5) and (7), we get

(8) L(α,ω,b)=∑i=1nαi−12∑i=1,j=1nYiYjαiαjXiTXj.

In (8), the value of *L*(*α*, *ω*, *b*) is only related to the parameter *α*. The training process of *α* can be solved by the sequential minimal optimization (SMO) algorithm [16–18].

## 3. C-SVM on the Penalty Parameter of the Error Term

The selection of the SVM interface is determinate according to the distribution of the support vectors. This means that a slight position change of one single support vector could lead to an obvious movement of the SVM interface. In another situation, if there is an instance in which a system outlier exists in the area of the opposite class, the SVM interface must be inflected so that it will no longer generate accurate classification results. Therefore, an error term is introduced to tolerate some erroneous instances in the opponent classification. In the C-support vector machine (C-SVM) model [19, 20], we use a nonnegative parameter *ς*_*i*_, for example, slack error term, which enables the geometric interval *s* < 1 between some erroneous instances and the SVM interface, according to (2). Slackening the restriction, we must rebuild the constraint function for the penalty of the outliers:

(9) min⁡ω,b⁡ 12||ω||2+C∑i=1nςis.t. Yi(ωTXi+b)≥1−ςi i=1,2,3,…,n   ςi≥0 i=1,2,3,…,n.

In (9), coefficient *C* is the penalty parameter of the error term, which is used to control the tolerance of the systematic outliers. A larger *C* value allows less outliers to exist in the opponent classification, or vice versa. Utilizing the Lagrangian function to calculate the extremum of (9), (5) can be rebuilt by

(10) L(α,ω,b,ς)  =12||ω||2+∑i=1nαi(1−Yi(ωTXi+b)−ςi)   +C∑i=1nςi−∑i=1nβiςi.

In (10), parameters *α*_*i*_ and *β*_*i*_ are the Lagrangian factors for the training instances and the systematic outliers, respectively. The extremum of *L*(*α*, *ω*, *b*, *ς*) can be acquired in correspondence with (6). Since *C* and *β* are not related to *ω* and *b* for SVM model, (9) can be subject to

(11) max⁡α⁡ W(α)=∑i=1nαi−12∑i=1,j=1nYiYjαiαj〈Xi,Xj〉s.t. 0≤αi≤C i=1,2,3,…,n     ∑i=1nαiYi=0.

When calculating (11) via the SMO process, one can adjust only two *α*_*i*_ at each iteration and consider the rest as the constants until it satisfies all Karush-Kuhn-Tucker (KKT) conditions [21–23]:

(12) α1Y1+α2Y2=−∑i=3nαiYi=ξ.

**Figure 1:**
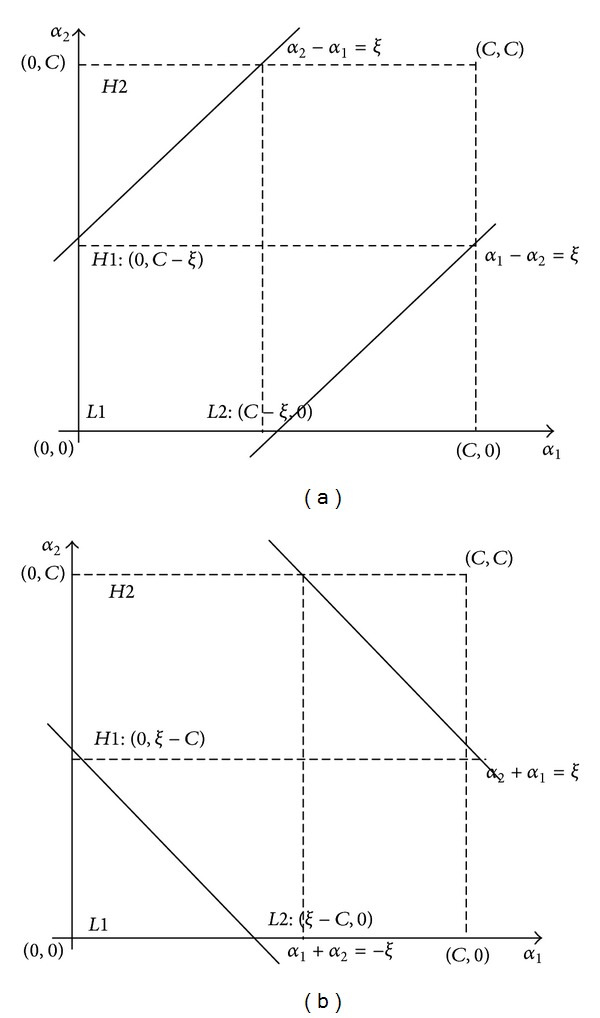
Parameter training process of SMO. (a) Adjustment of the parameter when Y1Y2 = 1. (b) Adjustment of the parameter when Y1Y2 = −1.

The output *Y* is labeled with +1 or −1 as the positive or the negative instance. Thereby, when *Y*_1_ *Y*_2_ = −1, (12) can be regarded as a line with gradient of 1: (*α*_1_ − *α*_2_ = *ξ* or *α*_2_ − *α*_1_ = *ξ*). When *Y*_1_ *Y*_2_ = 1, it can be regarded as a line with gradient of −1: (*α*_2_ + *α*_1_ = *ξ* or *α*_1_ + *α*_2_ = −*ξ*). When adjusting *α*_1_ and *α*_2_, the value of the parameters should satisfy the functions of the lines according to Figure 1. Meanwhile, they must be restricted within the square with length *C*, where *C* is the penalty parameter of the error term in (9). Therefore, when *Y*_1_ *Y*_2_ = −1,

(13) L=max⁡(0,α2−α1)H=min⁡(C,C+α2−α1);

otherwise,

(14) L=max⁡⁡(0,α2+α1−C)H=min⁡(C,α2+α1).

Continue the SMO process; set *K*_*ij*_ = 〈*X*_*i*_, *X*_*j*_〉:

(15) α2new=Y2(Y2−Y1+Y1ξ(K11−K12)+v1−v2)K11+K22−2K12

(16) α2new=α2old+Y2(E1−E2)η.

In (16), *E*_*i*_ is the dissimilarity between the real value of the model *v*_*i*_ = *ω* ^*T*^ *X*_*i*_ + *b* in (−*∞*, +*∞*) and the output of *Y*_*i*_ in [+1, −1]. By definition of *K*_*ij*_, *η* equals the square of the distance of the vectors; that is, *η* = ||*X*_*i*_−*X*_*j*_||^2^. As vector *X*_*i*_ follows a certain distribution, *η* is a constant in (16). The training process of the Lagrangian factors *α*_1_ and *α*_2_ is calculated by (15) and (16), and it is limited by (13) and (14). Integrated with KKT conditions, the final training process of *α*_2_ can be demonstrated by

(17) α2new,clipped={Lα2new≤Lα2newL<α2new<HHα2new≥H,

where *α*_1_ = (*ξ* − *α*_2_ *Y*_2_)*Y*_1_, *α*_1_^old^ = *ξ* − *Y*_1_ *Y*_2_ *α*_2_^old^, and *α*_1_^new^ = *ξ* − *Y*_1_ *Y*_2_ *α*_2_^new,clipped^. The training process of *α*_1_ can be finalized by

(18) α1new=α1old+Y1Y2(α2old−α2new,clipped).

The training process stops when all *α*_*i*_ values satisfy the KKT conditions:

(19) ai=0⟺Yivi≥10<ai<C⟺Yivi=1ai=C⟺Yivi≤1.

## 4. Optimization of the Penalty Parameter of the Error Term

Since there is no theoretical selection of the penalty parameter of the error term, grid-search is recommended on the value of *C* using cross-validation. Once the appropriate *C* is determined (e.g., *C* = 2^−5^, 2^−3^,…, 2^15^), the same value must be implemented on both positive and negative instances.

**Hypothesis 1 .**

**Figure 2:**
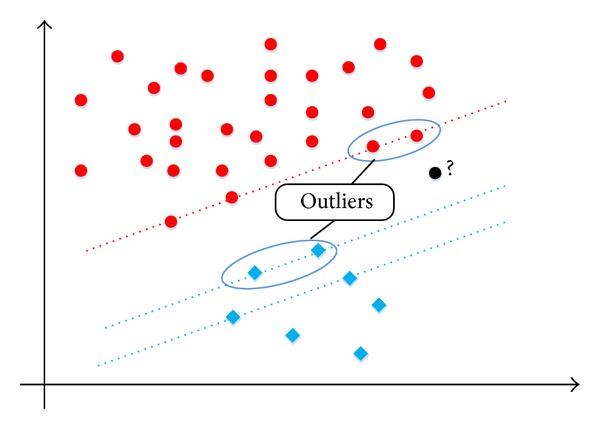
A heterogeneous distribution of the initial instances for C-SVM, Hypothesis 1: the number of the positive instances is much greater than the number of negative instances.

In Figure 2, the red dots represent positive instances, and the blue diamonds represent negative instances. Assume four instances (two positive and two negative) are outliers, which are circled by the black ellipses. The following will happen: since there is a large number of positive instances, deleting two of them as the support vectors may not change the position of the SVM interface. However, the same phenomenon does not occur with the negative instances. Deleting two negative support vectors will produce an obvious change in the position of the SVM interface. Thus, an unbeknown instance represented by a black dot will be erroneously classified to the positive set, which should have belonged to the negative set if *C* were not implemented in the SVM model.

According to the analysis above, we provide different values of *C* for positive instances and negative instances instead of a constant value of the penalty parameter for all nodes. Thus, (9) can be improved by

(20) min⁡ω,b⁡12||ω||2+C+∑i=1lςi+C−∑i=l+1l+mςis.t. Yi(ωTXi+b)≥1−ςi i=1,2,3,…,n   ςi≥0 i=1,2,3,…,n.

In (20), *l* presents all positive instances, and *m* denotes the negative instances. Since the positive instances can tolerate more system outliers due to the large number of instances, *C* ^+^ can be assigned a smaller value than *C* ^−^.

**Hypothesis 2 .**

**Figure 3:**
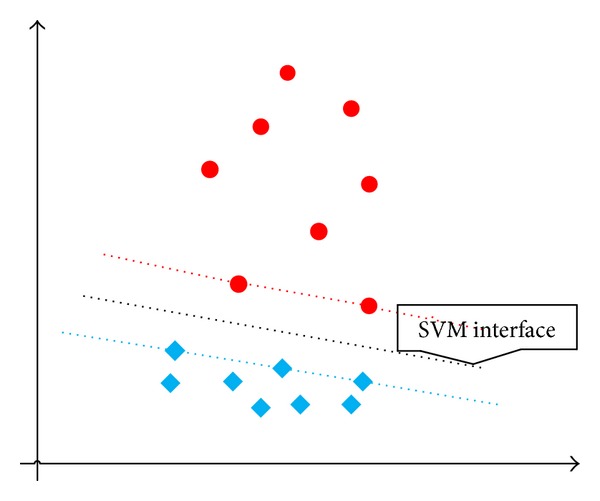
Heterogeneous distribution of the initial instances for C-SVM, Hypothesis 2: when the number of positive instances is equal to the number of negative instances, the positive instances account for a larger area.

In Figure 3, the number of positive instances is equal to the number of negative instances, but the negative instances can tolerate more system outliers due to the initial distribution of the data.

**Hypothesis 3 .**

**Figure 4:**
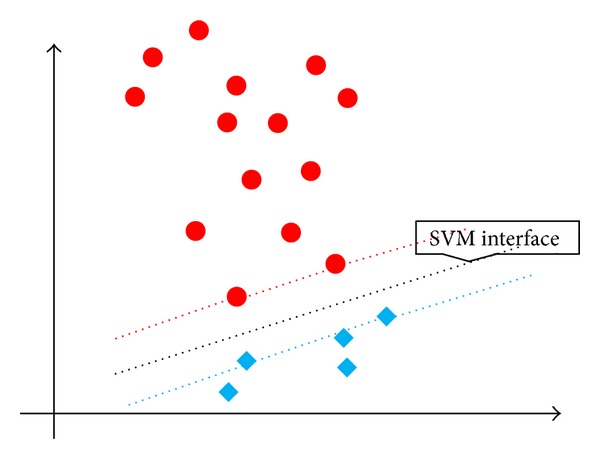
Heterogeneous distribution of the initial instances for C-SVM, Hypothesis 3: when the number of positive instances is larger than the number of negative instances, the positive instances account for a larger area.

In Figure 4, the number of positive instances is even larger than the number of negative instances, but the penalty parameter for the positive instances can be stricter than for the negative instances. Therefore, *C* ^+^ must be assigned a larger value in Hypothesis 2 than in Hypothesis 3.

Integrated with all the hypotheses, we find that the proportion of *C* ^+^ and *C* ^−^ is relevant to the number of positive and negative instances and the distribution of the initial data samples. Therefore, we propose a density-based, penalty parameter optimization of the error term:

(21) D+=|max⁡((ωTXi+b)/||ω||)−min⁡((ωTXi+b)/||ω||)|lD−=|min⁡((ωTXi+b)/||ω||)−max⁡((ωTXi+b)/||ω||)|m.

**Figure 5:**
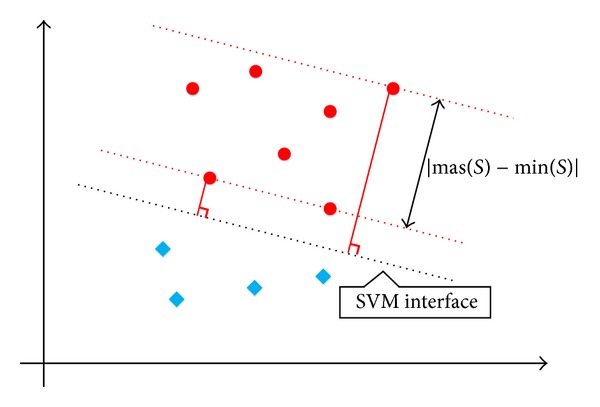
Density-based, parameter-weights optimization on the distribution of a heterogeneous dataset for C-SVM.

In (21), *D* ^+^ and *D* ^−^ present the sample density of the positive instances and the negative instances, respectively. The larger the value of *D* is, the smaller the sample density is, and, thus, a smaller *C* can be assigned. According to Figure 5, the density of the corresponding instances is decided by the distance between the remotest node and the nearest node from the SVM interface divided by the number of instances.

## 5. Experiments

We chose a dataset from the official website of LIBSVM, which contains many classifications, regressions, and multilabel datasets stored in LIBSVM format. Many are from UCI, Statlog, StatLib, and other collections [24]. The data groups used in our experiments are listed in Table 1.

In order to evaluate the accuracy of our proposed algorithm, we optimized the C-SVM model based on LIBSVM tools [14] using linear kernel function *ω* ^*T*^ *X* + *b*. The comparative tests are set by (1) the uniform *C* for both instances as traditional C-SVM, (2) the *C* ^+^ and *C* ^−^ that correspond to the ratio of the positive instance number and the negative instance number, and (3) the *C* ^+^ and *C* ^−^ that correspond to our proposed, density-based, penalty parameter optimization.

Aiming at testing whether the proposed algorithm has a positive performance under all circumstances, we simply assigned *C* the values of 0.5, 1, 10, 50, and 100 instead of doing the grid-search. In our proposed optimization, (21) can provide only the proportions of *C* ^+^ and *C* ^−^, but not the exact values. Therefore, we used

(22) C+=C+Δ (C=0.5,1,…,100)C−=C−Δ (C=0.5,1,…,100)C+C−=D+D−.

For comparative test 2, the proportion of the *C* ^+^ and *C* ^−^ was decided by the number of positive instances *N* ^+^ and the number of negative instances *N* ^−^:

(23) C+=C+Δ (C=0.5,1,…,100)C+=C+Δ (C=0.5,1,…,100)C+C−=N−N+.

In our proposed algorithm, the SVM interface is unknown before the classification. In order to calculate the density of the corresponding class, we first implement a traditional C-SVM and confirm the position of the SVM interface. In this way, the densities of the positive and negative instances can be computed via (21), and then *C* ^+^ and *C* ^−^ eventually can be determined by (23).

We evaluated the accuracy of our proposed algorithm via precision, recall, and *F*-measure. The precision rate was the number of correctly classified instances divided by the number of total instances.  Table 2 shows the experimental results of the precision rate of the different algorithms for different *C* values, where a1a-1, a1a-2, and a1a-3 present the traditional C-SVM, improved C-SVM on number proportion (23), and our proposed, density-based C-SVM (22), respectively.

Recall rate indicates the number of the right classified positive instances by the number of the total positive instances in the testing set.  Table 3 shows the experimental results of the recall rates provided by the different algorithms different values of *C*.

Table 1 indicates that the size of the testing set of w1a was 47,272, which was composed of 1,407 positive instances and 45,865 negative instances. For this distribution, we predict all unknown inputs as the negative instances. In this way, all of the 45,865 negative instances can be classified correctly with the precision of 97.02%. Therefore, the recall rate is of great importance as a supplementary measure. In a1a, a2a, a3a, and as a4a datasets, the size of the negative instances is about double that of the positive instances. Method 2 (number proportion-based optimization) sacrifices the precision rate in an acceptable range, but it improves the recall rate in a large scale. In Method 3 (our proposed density-based C-SVM), 12 groups in 20 experiments had slightly decreased precision rates, while the other eight groups successfully enhanced it. All 20 experiments by Method 3 improved the recall rate, but not to the extent that Method 2 did.

In w1a, w2a, w3a, and w4a datasets, the size of the negative instances was many times greater than that of the positive instances. Our proposed method indicated that there were obvious advantages in both precision rate and recall rate. Traditional C-SVM has a high precision rate, but it performs poorly with respect to recall rate. Method 2 improved the recall performance and decreased the precision rate, which was similar to the findings of previous experiments. Method 3 enhanced the precision rate to a greater extent than traditional C-SVM, and it simultaneously improved the recall rate over that of Method 2.

The *F*-measure is a comprehensive evaluation of both precision and recall. In (24), beta is the parameter that adjusts the weights between the precision rate and the recall rate. When we consider precision more important, the value of beta should be > 1. On the contrary, in some cases, such as alarming or warning, the recall rate is significant in determining all of the potential risks. Thus, the value of beta should be < 1:

(24) $$\begin{array}{c} F\text{-measure}=\frac{\left({\left(\text{beta}\right)}^{2}+1\right)\times \text{PRE}\times \text{REC}}{{\left(\text{beta}\right)}^{2}\times \text{PRE}+\text{REC}}. \end{array}$$

**Figure 6:**
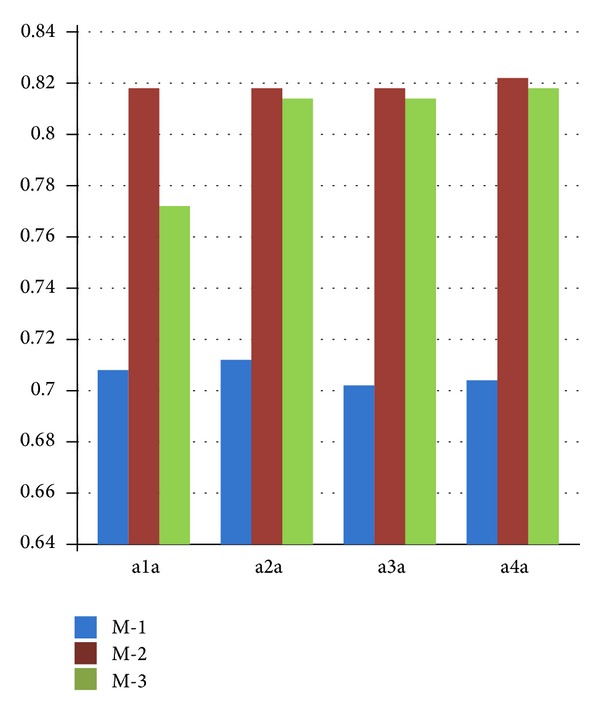
Comparison of the *F*-measure among traditional C-SVM (M-1), number proportion-based optimization (M-2), and density-based optimization (M-3) via datasets in which the size of the negative instances was several times greater than that of the positive instances.

**Figure 7:**
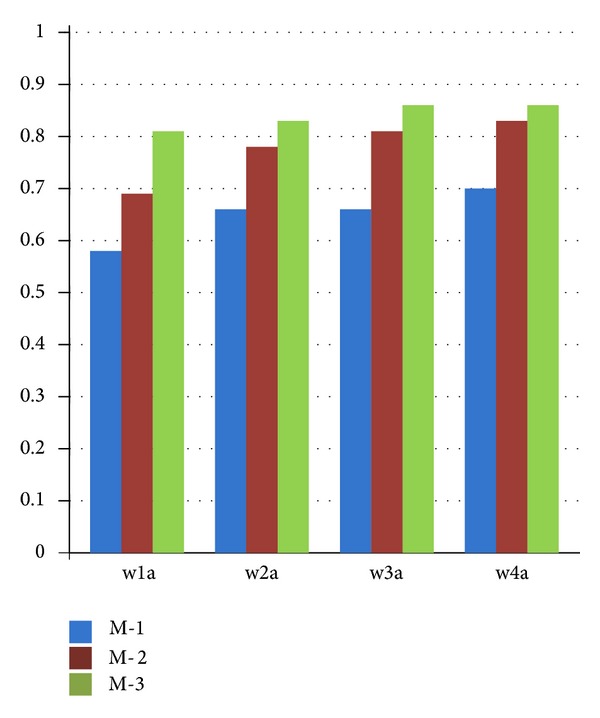
Comparison of the *F*-measure among traditional C-SVM (M-1), number proportion-based optimization (M-2), and density-based optimization (M-3) via datasets in which the size of the negative instances was far greater than that of the positive instances.

Table 4 provides the evaluation results by *F*-measure with beta = 1. Figures 6 and 7 explicitly demonstrate that the comparisons among M-1 (traditional C-SVM), M-2 (number proportion-based C-SVM optimization), and M-3 (density-based C-SVM optimization). Each statistical result was obtained by the average of one certain data group for *C* = 0.5, 1, 10, 50, and 100.

Figure 6 shows datasets a1a, a2a, a3a, and a4a, in which the size of the negative instances was several times greater than that of the positive instances. Both M-2 and M-3 can generate better *F*-measure evaluations than M-1, traditional C-SVM. Concerning the *F*-measure, M-2 performs even better, but, in doing so, systematic precision was sacrificed in order to achieve better recall. Our proposed M-3 minimizes the losses of systematic precision and evidently enhances the *F*-measure to a greater extent than M-1.

Figure 7 shows datasets w1a, w2a, w3am, and w4a, in which the size of the negative instances is far greater than that of the positive instances. M-3 had the best results for precision, recall, and *F*-measure. Therefore, for the given data distribution, our proposed density-based C-SVM optimization provided a remarkable advantage for the classification of data.

## 6. Conclusions

In this paper, we presented density-based penalty parameter optimization in C-SVM algorithm. In traditional C-SVM, as the penalty parameter of the error term, *C* is used to control the tolerance of the systematic outliers. A larger value of *C* allows less outliers to exist in the opponent classification. Grid-search is generally implemented in the computation of the values of *C*. In order to enhance the accuracy of the algorithm, LIBSVM sets different values of *C* for positive and negative slack error terms based on the number proportion of the positive and negative instances. The principle of number proportion-based C-SVM optimization is that the weight of each instance is decided by the possibility that this instance itself is a system outlier and by the extent to which it will lead the change in the position of the SVM interface. Motivated by this idea, our proposed density-based penalty parameter optimization is more integrated consideration that includes the sizes of the positive and negative instances and takes the distribution of those instances into account. We implemented our experiments in the standard datasets for classifications. The results of the evaluation indicated that number proportion-based C-SVM optimization normally deserves a better *F*-measure, but it enhances the systematic recall in a large scale while simultaneously decreasing the systematic precision. Compared with number proportion-based C-SVM optimization, our proposed density-based method improved the systematic recall and maintained systematic precision according to traditional C-SVM. Our proposed density-based method demonstrated outstanding performance on both precision and recall, especially for datasets in which the number of negative instances was far greater than the number of positive instances.

## Figures and Tables

**Table 1:** Standard dataset for classification.

Name	Type	Class	Training size	Testing size	Feature
a1a	Classification	2	1,605	30,956	123
a2a	Classification	2	2,265	30,296	123
a3a	Classification	2	3,185	29,376	123
a4a	Classification	2	4,781	27,780	123
w1a	Classification	2	2,477	47,272	300
w2a	Classification	2	3,470	46,279	300
w3a	Classification	2	4,912	44,837	300
w4a	Classification	2	7,366	42,383	300

**Table 2:** Systematic precision at different parameter values (%).

Model	*C* = 0.5	*C* = 1	*C* = 10	*C* = 50	*C* = 100
a1a-1	84.03	83.82	83.77	83.69	83.64
a1a-2	77.41	78.65	78.56	78.65	78.72
a1a-3	83.92	83.31	83.26	83.11	83.10
a2a-1	84.60	84.28	84.02	83.98	83.95
a2a-2	76.89	77.27	77.26	77.19	77.20
a2a-3	84.47	84.30	84.04	83.92	83.88
a3a-1	84.50	84.32	84.08	84.07	84.07
a3a-2	77.53	77.88	77.89	77.83	77.80
a3a-3	84.37	84.35	84.11	84.08	84.07
a4a-1	84.29	84.25	84.18	84.06	84.07
a4a-2	78.49	79.22	79.13	79.20	79.16
a7a-3	83.96	84.08	84.12	84.23	84.11
w1a-1	97.56	97.74	97.46	96.75	96.56
w1a-2	95.10	96.10	94.94	94.25	94.66
w1a-3	97.62	97.84	97.53	97.34	97.21
w2a-1	97.86	98.07	97.53	97.27	97.07
w2a-2	94.88	96.03	95.78	94.86	94.90
w2a-3	98.12	98.21	97.92	97.63	97.58
w3a-1	97.83	98.29	98.02	97.84	97.83
w3a-2	95.27	96.22	96.01	95.51	95.62
w3a-3	97.91	98.24	98.36	98.07	98.02
w4a-1	98.01	98.39	98.26	98.07	97.95
w4a-2	95.58	96.52	96.50	96.15	96.22
w4a-3	98.01	98.42	98.38	98.27	98.26

**Table 3:** Systematic recall for different parameter values (%).

Model	*C* = 0.5	*C* = 1	*C* = 10	*C* = 50	*C* = 100
a1a-1	60.07	60.60	61.46	61.50	61.46
a1a-2	87.21	85.87	85.14	84.99	84.97
a1a-3	73.02	72.73	72.16	71.54	71.52
a2a-1	59.17	62.04	62.29	62.61	62.77
a2a-2	88.10	87.54	86.34	86.22	86.19
a2a-3	79.37	79.22	78.94	78.73	78.56
a3a-1	57.64	60.58	60.70	60.70	60.71
a3a-2	87.28	86.39	85.84	85.67	85.75
a3a-3	78.93	79.01	78.54	78.33	78.28
a4a-1	58.39	60.20	60.90	60.90	60.92
a4a-2	86.61	85.69	85.57	85.60	85.65
a4a-3	80.01	79.56	79.34	79.42	79.38
w1w-1	19.97	50.39	49.82	45.98	46.41
w1w-2	66.95	59.51	50.82	47.46	47.69
w1w-3	70.42	70.13	68.24	67.03	67.12
w2w-1	31.41	51.24	56.27	57.14	56.49
w2w-2	73.03	70.12	65.01	61.52	60.64
w2w-3	72.76	72.53	71.88	72.02	70.93
w3w-1	29.42	53.97	58.01	56.59	58.23
w3w-2	77.47	73.05	68.34	65.64	63.47
w3w-3	77.12	76.87	76.31	75.54	75.65
w4w-1	36.18	55.50	60.73	61.84	62.07
w4w-2	77.28	73.71	70.31	68.17	67.46
w4w-3	77.37	76.52	76.43	75.12	74.83

**Table 4:** Systematic *F*-measure at different parameter values (%).

Model	*C* = 0.5	*C* = 1	*C* = 10	*C* = 50	*C* = 100
a1a-1	70.06	70.34	70.90	70.90	70.85
a1a-2	82.02	82.10	81.72	81.70	81.73
a1a-3	78.09	77.66	77.31	76.89	76.88
a2a-1	69.63	71.47	71.54	71.74	71.84
a2a-2	82.12	82.09	81.55	81.45	81.44
a2a-3	81.84	81.68	81.41	81.24	81.13
a3a-1	68.53	70.51	70.50	70.50	70.51
a3a-2	82.11	81.91	81.67	81.56	81.58
a3a-3	81.56	81.59	81.23	81.10	81.07
a4a-1	69.09	70.29	70.78	70.77	70.79
a4a-2	82.35	82.33	82.22	82.28	82.27
a4a-3	81.94	81.76	81.66	81.75	81.68
w1w-1	33.16	66.50	65.94	62.34	62.70
w1w-2	78.58	73.81	66.20	63.40	63.43
w1w-3	81.82	81.70	80.30	79.39	79.41
w2w-1	47.56	67.31	71.37	71.99	71.42
w2w-2	82.53	81.05	77.45	74.63	74.00
w2w-3	83.56	83.44	82.90	82.89	82.15
w3w-1	45.23	69.68	72.88	71.70	73.01
w3w-2	85.45	83.05	79.85	77.81	76.30
w3w-3	86.28	86.25	85.94	85.34	85.39
w4w-1	52.85	70.97	75.06	75.85	75.99
w4w-2	85.46	83.59	81.35	79.78	79.31
w4w-3	86.48	86.10	86.03	85.15	84.96

## References

[1] Hosmer JR, David W, Stanley L, Rodney X (2013). *Applied Logistic Regression*.

[2] Darroch JN, Ratcliff D (1972). Generalized iterative scaling for log-linear models. *Annals of Mathematical Statistics*.

[3] Lin C-J, Weng RC, Keerthi SS Trust region Newton methods for large-scale logistic regression.

[4] Lin CJ, More JJ (1999). Newton's method for large bound-constrained optimization problems. *SIAM Journal on Optimization*.

[5] Mangasarian OL (2002). A finite Newton method for classification. *Optimization Methods & Software*.

[6] Nash SG (2000). A survey of truncated-Newton methods. *Journal of Computational and Applied Mathematics*.

[7] Kao WC, Chung KM, Sun CL, Lin CJ (2004). Decomposition methods for linear support vector machines. *Neural Computation*.

[8] Joachims T Training linear SVMs in linear time.

[9] Schölkopf B, Burges CJC, Smola AJ (1998). *Advances in Kernel Methods—Support Vector Learning*.

[10] Shalev-Shwartz S, Singer Y, Srebro N Pegasos: primal estimated sub-Gradient solver for SVM.

[11] Boser BE, Guyon IM, Vapnik VN Training algorithm for optimal margin classifiers.

[12] Hsieh CJ, Chang KW, Lin CJ, Keerthi SS, Sundararajan S A dual coordinate descent method for large-scale linear SVM.

[13] Fan R-E, Chang K-W, Hsieh C-J, Wang X-R, Lin C-J (2008). LIBLINEAR: a library for large linear classification. *Journal of Machine Learning Research*.

[14] Chang CC, Lin CJ LIBSVM: a library for support vector machines. http://www.csie.ntu.edu.tw/~cjlin/libsvm/.

[15] Li H, Guan XH, Zan X (2003). Network intrusion detection based on support vector machine. *Journal of Computer Research and Development*.

[16] Platt J (1998). Sequential minimal optimization: a fast algorithm for training support vector machines. *Advances in Kernel Methods: Support Vector Learning*.

[17] Platt JC (1999). *Fast Training of Support Vector Machines using Sequential Minimal Optimization*.

[18] Cao LJ, Keerthi SS, Ong CJ (2006). Parallel sequential minimal optimization for the training of support vector machines. *IEEE Transactions on Neural Networks*.

[19] Li H, Guan XH, Zan X, Han CZ (2003). Network intrusion detection based on support vector machine. *Journal of Computer Research and Development*.

[20] Liu S, Jia CY, Ma H A new weighted support vector machine with GA-based parameter selection.

[21] Kuhn M (2006). *The Karush-Kuhn-Tucker Theorem*.

[22] Qi L, Jiang H (1997). Semismooth Karush-Kuhn-Tucker equations and convergence analysis of Newton and quasi-Newton methods for solving these equations. *Mathematics of Operations Research*.

[23] Bach FR, Lanckriet GRG, Jordan MI Multiple kernel learning, conic duality, and the SMO algorithm.

[24] Hsu CW, Chang CC, Lin CJ A practical guide to support vector classification. https://www.cs.sfu.ca/people/Faculty/teaching/726/spring11/svmguide.pdf.

